# Determination of Annual Plasma Hormone Levels Associated with Reproduction in Long-Day Breeding Domestic Geese

**DOI:** 10.3390/ani11082363

**Published:** 2021-08-10

**Authors:** Małgorzata Gumułka, Nataly Avital-Cohen, Israel Rozenboim

**Affiliations:** 1 Department of Animal Reproduction, Anatomy and Genomics, University of Agriculture in Kraków, Al. Mickiewicza 24/28, 30-059 Kraków, Poland; 2 Department of Animal Sciences, Robert H. Smith Faculty of Agriculture, Food and Environment, Hebrew University of Jerusalem, P.O. Box 12, Rehovot 76100, Israel; Natalie.Avital@mail.huji.ac.il (N.A.-C.); rully.r@mail.huji.ac.il (I.R.)

**Keywords:** reproduction, geese, prolactin, thyroid hormones, gonadal steroids

## Abstract

**Simple Summary:**

In domestic birds, breeding practices and optimisation of the microenviroment and nutrition ensure egg production throughout the entire year. However, domestic geese experience an annual cycle of reproductive quiescence and recrudescence. Thus, patterns of reproductive hormones related to the initiation and termination of the breeding–laying period between the sexes seems to be especially important. This paper presents annual patterns of prolactin (PRL), triiodothyronine (T3), thyroxine (T4), testosterone (T), progesterone (P4), and estradiol (E2) in ganders and female geese. Long-day breeding Zatorska geese kept in controlled commercial conditions experienced periods with elevated plasma PRL levels in both sexes post-breeding and during the second half of the breeding–laying period. Increased plasma PRL levels by the end of the breeding–laying period were detected earlier in ganders than in female geese. Annual patterns of thyroid hormones (THs) were partially in agreement with existing theories on the specific role of THs in termination of breeding, which is permissive rather than causal. It may be suggested that ganders terminate their breeding–laying period one month earlier than female geese. These results may be useful in the manipulation of the endocrine axis to extend the duration of seasonal hatching egg production.

**Abstract:**

This paper examines the dynamics of circulating hormone changes connected with reproduction in geese during the annual period related to gonad morphometry. One hundred geese were examined. The levels of prolactin (PRL), triiodothyronine (T3), thyroxine (T4), testosterone (T), progesterone (P4) and estradiol (E2) were estimated. In both sexes, PRL level patterns fit a quadratic trend with elevations in the post-breeding and the second half of the breeding–laying periods. During these periods, differences in the PRL level between sexes were noted. In ganders, increased PRL levels during the laying period occurred earlier compared to in female geese. Cubic trends for T and E2 in ganders and quadratic for T, P4, and E2 in female geese were observed. PRL was negatively correlated with T in both sexes and with P4 and E2 in female geese. A higher level of T3 and variation in T4 in ganders with a quartic trend in ganders vs. a quadratic in female geese were noted. Patterns of PRL, T, and E2 suggested that the breeding–laying period in ganders may be shorter than in female geese. These findings will be used to explore experimental manipulations of the endocrine axis to increase synchronisation of both sexes.

## 1. Introduction

Seasonal reproduction in free-living birds results in the production of offspring during the optimum time for survival. Photoperiodic changes are the major cue used by birds in determining the timing and duration of the breeding period [1,2,3]. Overall, photoperiod induced thyroid hormones (THs) activation or deactivation in the hypothalamus, which plays a role in the regulation of the hypothalamic–pituitary–gonadal axis (HPG) [4]. Photostimulation causes the upregulation of THs production via TSH–induced activation of TH-activating enzyme that converts the precursor thyroxine (T4) to bioactive triiodothyronine (T3). Locally produced T3 promotes the release of the neuropeptide gonadotropin-releasing hormone (GnRH) [5]. Hypothalamic GnRH and vasoactive intestinal peptide (VIP) induce the synthesis and secretions of gonadotropins (luteinizing hormone [LH] and follicle-stimulating hormone [FSH]) and prolactin (PRL) by the pituitary gland. Gonadotropins stimulate gametogenesis and sex steroid synthesis in the gonad [2]. Moreover, plasma changes in PRL [6] and THs [7] levels are related to natural moult occurring by the end of breeding season. At this phase, photorefractoriness occurs, and HPG axis activity is terminated, which relates to regression of the gonads [4].

In domestic birds, breeding practices and optimisation of the microenvironment and nutrition ensure egg production all through the year. However, domestic geese experience an annual cycle of reproductive quiescence and recrudescence [8]. Commercially used geese breed in Europe’s climatic zone are long-day breeders. Breeding can be distinguished to 4 stages: post-breeding, non-breeding, pre-breeding, and the breeding–laying period [9]. Long-day breeding geese, kept under a controlled photoperiod regime, produced only 70–80 hatching eggs per season [10]. In addition, by the end of the laying period, a reduction in fertility was observed [11]. From a breeding point of view, study on the seasonality of reproductive endocrinology of domestic geese is justifiable in the context of developing methods to control fertility. Understanding the exact annual pattern of hormones related to initiation and termination of the breeding–laying period in geese may be useful to manipulation of the duration of the egg production period.

To date, seasonal patterns of circulating gonadal steroids [12] and THs, PRL, and LH [13] have been reported in long-day breeding European geese. Moreover, Péczely et al. [7] showed the level of gonadal and adrenal steroids and THs in commercial crosses of European geese. Also, in a previous study, patterns of chosen hormones connected with reproduction in long-day breeding Chinese geese kept in natural [14] or different controlled photoperiod conditions [15,16] were noted. Additionally, endocrine mechanisms of seasonal reproduction with circulating hormone changes in short-day Chinese geese were investigated [17,18]. Recently, research on short-day geese plasma hormone levels associated with natural daylight reproduction was presented [19]. 

However, in the available literature there are no data showing simultaneously determined changes in reproductive hormones in long-day ganders and female geese kept in the same microenvironmental and photoperiod commercial conditions in one season. In addition, in studies performed on European geese [7,13] data were collected from both sexes of geese only during a chosen time of the annual cycle or geese were kept under natural environmental and breeding conditions [20]. Furthermore, in biparental species, reproductive success depends on the degree of hormonal compatibility among parents [21]. In greylag geese (*Anser anser*), a positive within-pair T co-variation (TC) among pair partners related to long-term reproductive success was noted [22]. In polygynous domestic geese, high degrees of within-pair TC also occurred but ranged from positive to non-correlated [20]. In studies on wild birds, special effort is also put on PRL as a “parental hormone”, which interacts with others to affect breeding success [23].

Current interest is high in developing methods to control the effectiveness of egg production in flocks of endangered geese species. Zatorska goose is kept as a part of a genetic resource conservation program [24] and is registered in the FAO World Watch List [25]. To date, changes in the concentration of reproductive hormones have been presented only in Zatorska ganders in connection with the study of mating activity [11], semen quality [26] and in relation to changes in artificial photoperiod conditions [27].

Therefore, we hypothesise that during the annual period, synchronised changes in reproductive hormones connected with gonadal response in Zatorska goose will occur. Thus, it seems reasonable to undertake an additional investigation attributable to the orchestration of annual reproductive hormone profiles in long-day breeding geese managed in controlled environmental conditions with a short-day light regime. The objectives of this study were (1) to determine the changes in plasma concentrations of PRL, THs and gonadal steroid hormones in Zatorska geese during post-breeding, non-breading, pre-breeding and photoperiodically induced laying periods with connection to gonads morphometry and (2) to examine the similarity of hormone trend patterns between ganders and female geese during the annual period in a polygynous flock structure.

## 2. Materials and Methods

### 2.1. Experimental Birds and Management 

All procedures were approved by the First Local Ethical Committee on Animal Testing of the Jagiellonian University in Krakow, Poland (PL). One hundred 2-year-old native Zatorska geese (ZD–1 strain) were individually marked and housed in a commercial light-proof building (1.0 bird/m^2^) located at the Experimental Station of the Agricultural University in Cracow–Poland. The breeding flock, consisting of 10 males and 40 females (sex ratio: 1 ♂: 4 ♀) kept on deep litter with nests for individual laying (breeding–laying period). During the annual period birds were managed as a flock in multi-male and multi-female social structure in accordance with commercial breeding rules. In addition, in two separate pens, 25 males and 25 females were managed. 

The management practices complied with the recommended rules for reproductive flocks of geese [9]. During the non-breeding period geese were fed under a semi-controlled feeding regime with oat and wheat grain as well as carrots and hay. During the breeding–laying period geese were fed daily with 190 g layer–breeding mixture (16% CP/kg and 2640 kcal ME_N_/kg) per day and 50 or 70 g of oat and wheat grain, respectively. Birds had free access to water.

**Figure 1:**
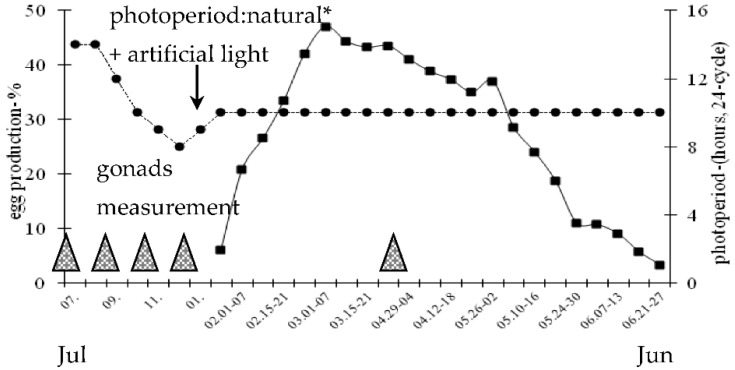
Weekly egg production (mean ratio–%) of 2-year-old Zatorska female geese (*n* = 40) and photoperiod conditions during the annual breeding period. * 50°03′ N/19°57′ E. Egg production was calculated based on individual laying control. Gonads measurement: post-breeding (July), non-breeding (September), pre-breeding (November), onset of laying (January), breeding–laying (April) stages.

During the annual period, both natural and artificial photoperiods were utilized (Figure 1). From July to September a natural photoperiod of about 12–14 h of light was used. Then birds were transferred to an artificial photoperiod of 10 L: 14 D, which was reduced to 8 L: 16 D in December. At the beginning of January, the daily photoperiod was gradually increased (+ 30 min/week) to 10 L: 14 D (07:00 a.m. to 05:00 p.m., 24 h light/dark cycle) at the end of the month. This short-day photoperiod was applied to the end of the reproduction period (i.e., to June).

### 2.2. Measurement Parameters

Daily egg production was recorded, and the curve was prepared. For each individual, the sexual reactivation (seasonal reactivation of the sexual maturity) of female geese was defined as the number of days from the 1st of January up to the day the first egg was laid (according to rules recommended for reproductive geese flocks in Poland). 

Blood samples were collected twice a month from 10 ganders and 19 female geese kept as a breeding flock (10 males and 40 females). Females were selected at random. Blood samples were collected into heparinized test tubes from the brachial vein in the morning (10:00 a.m. to 12:00 a.m.). Plasma was separated by centrifugation (laboratory centrifuge MPW 251, MPW Med. Instruments, Warsaw, Poland; 1500× *g*/10 min) and stored at −20 °C for subsequent analysis. 

In addition, characteristic stages of the geese’s annual breeding period were chosen (i.e., post-breeding, non-breeding, pre-breeding, onset of laying and breeding–laying). Annual stages were determined based on reproduction performance of female geese (i.e., eggs laying pattern) obtained under the regulated conditions of daylight and feeding in commercial practice. During these five cycles, birds kept in isolated pens (5 males and 5 females) were selected at random and slaughtered. Body weight was recorded as well as testis, ovary and oviduct weights.

### 2.3. Hormone Analysis 

Concentration of ovarian steroid hormones progesterone (P4), estradiol (E2), testosterone (T) and PRL in plasma samples were measured by an enzyme-linked immunosorbent assay (ELISA). A previously presented method for P4, E2, T [28] was performed, which was validated for plasma from ganders [26]. Steroid hormones were extracted from 0.5 mL of plasma with 5 mL of diethyl ether. Recovery after extraction was 90% for P4, E2, and T. Dilutions of primary antibodies and tracers were 1:5000 and 1:50, 1:160,000 and 1:160, and 1:320,000 and 1:320 for P4, E2, T, respectively. All samples were analysed in duplicate, and for every other plate, a separate standard curve was determined. The range of the assay was 0.78 to 400 pg/mL and the intraassay CV was 5% for P4, E2, T. Cross-reactivity of antitestosterone (ICN 647381, Irvine, CA, USA) was determined with dihydrotestosterone, androstenedione, dehydroepiandrosterone, estradiol, progesterone, and corticosterone. Only dihydrotestosterone had more than 5% cross-reactivity (55.3%). Cross-reactivity of antitestradiol (ICN 614051, Irvine, CA, USA) was determined with estrone, estriol, dihydrotestosterone, androstenedione, dehydroepiandrosterone, testosterone, progesterone, and corticosterone and was found to be less than 2%. According to the manufacturer’s data, cross-reactivity of antiprogesterone (A1405, AbKem Iberia S.L., Vigo, Spain) with gonadal and nongonadal steroids was less than 5%. 

Plasma PRL was assayed by competitive ELISA with the use of biotinylated PRL tracers according to a method described by Rochester et al. [29] which was previously validated for plasma from ganders [26,27]. The minimal detectable dose was 4 ng/mL. Absorbance at 405 nm was read in a Tecan Sunrise Microplate reader (Tecan Group Ltd., Mannedorf, Switzerland). The assay was conducted in duplicates and for every other plate, a separate standard curve was determined. The intraassay CV was 7%.

The concentrations of thyroxine (T4) and triiodothyronine (T3) in the plasma were determined by radio immunological tests RIA using BRAHMS^®^ kits (BRAHMS GmbH—part of Thermo Fisher Scientific, Hennigsdorf, Germany) supplied by Nobipharm (Warsaw, Poland). The lowest limits of sensitivity for T4 and T3 were 0.08 and 1.3 ng/mL, while the intra-assay coefficients of variation were 5.8% and 6.2%, respectively. Plasma concentrations of all hormones were determined in one analysis to avoid inter-assay variation.

### 2.4. Statistical Analyses 

The variables were examined for normality using Kolmogorov-Smirnov and Shapiro-Wilk tests. Also, the homogeneity of the variances was assessed using the Brown-Forsythe test. Data for the body weight and gonads characteristics were analysed by Kruskal-Wallis H test and significance of the difference for medians between stages of the annual period were analysed by Dunn’s test. For differences in median number of SYF and LYF follicles in ovary Mann-Whitney U test was used. The results of the hormone concentrations were log–transformed prior to analysis. After log-transformation the data obtained normal distribution. Hormone levels depending on the sex and the month of the annual period were examined by two-way analysis of variance. In a post-hoc test, gender contrast analysis (differences between sexes in each month) and a month-to-month contrast analysis were performed by testing linear, quadratic, cubic, and quartic trends. Relationships between various hormone levels during the annual period were analysed by estimating Pearson’s linear correlation coefficients. Statements of statistical significance were based on *p* < 0.05, *p* < 0.01 and *p* < 0.001. Untransformed, raw data are presented as means for hormone concentrations. Calculations were performed with PQStat Software (PQStat Software, Poznan, Poland) and NCSS 2020 Software (NCSS, LLC, Kaysville, UT, USA).

## 3. Results

### 3.1. Reproduction Results 

Female geese sexual reactivation, calculated as number of days from 1 January, was 37.0 ± 1.2 days. The age of female geese at sexual reactivation was 640 days. Laying percentage from 18 January to 27 June was 26.7% (Figure 1). After 5 weeks from the onset of egg production the peak of laying (March, 47.0%) was noted. During the period from March to the onset of April laying rate remained relatively constant at a level of about 43.0%. Then eggs production slowly decreased to the rate of about 28.0% by the onset of May. In the next period a sharp decrease in laying rate was observed to the level of 3.3% by the end of June. The mean number of eggs was 41 ± 1.4/goose/season (January–June). Egg weight was 168.9 ± 2.3 g.

In the period post-breeding (July) to breeding-laying (April) the ganders’ body weight median ranged from 4891 g to 6597 g (Table 1), and female geese body weights median ranged from 4770 g to 5709 g (Table 2). 

During the annual period, differences in testis and ovary weight were observed (Table 1 and Table 2). The right testis median weight was higher (*p* < 0.05) at the onset of laying (January) compared to non-breeding (September) stage. The left testis weight was higher (*p* < 0.05) at the onset of laying and breeding–laying (April) than in non-breeding stage. Median weight of the ovary was higher (*p* < 0.05) in onset of laying and breeding–laying than in non- and pre-breeding stages. In active reproduction stages an additional class of follicles i.e., small yellowish follicle and large yellow follicle were noted.

### 3.2. Plasma Concentration of Hormones in Ganders and Female Geese 

#### 3.2.1. PRL Concentration 

**Figure 2:**
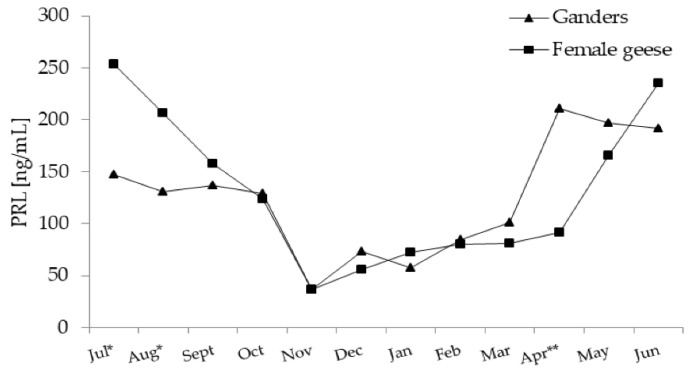
Plasma concentration of PRL (prolactin) in 2-year-old Zatorska ganders (*n* = 10) and female geese (*n* = 19) during the annual period. Breeding flock (10 ganders and 40 female geese; natural mating). Reproduction period: January–June. Photoperiod during reproduction: short-day (SD) 10 L:14 D. Data are means presented in monthly intervals. Annual pattern of PRL in ganders and female geese fit a quadratic trend (*p* < 0.01). The months with different superscripts indicate significant differences between means for sex—ganders vs. female geese * *p* < 0.05; ** *p* < 0.01).

In both sexes, annual PRL concentration patterns fit a significant (*p* < 0.01) quadratic trend, which was concave upward (Figure 2). 

In the period from July–November, PRL levels decreased and then, as the reproduction season progressed (January–March), slowly increased. A sharp increase in PRL level was noted in ganders in April and in female geese in May. 

In July and August PRL concentration was higher (*p* < 0.05) in female geese compared to ganders. While in April PRL, concentration was higher (*p* < 0.01) in ganders than in female geese. A significant interaction (*p* < 0.01) between the sex and month factors during the annual period was demonstrated.

#### 3.2.2. Thyroid Hormones Concentrations—T3 and T4 

##### T3 Concentration

**Figure 3:**
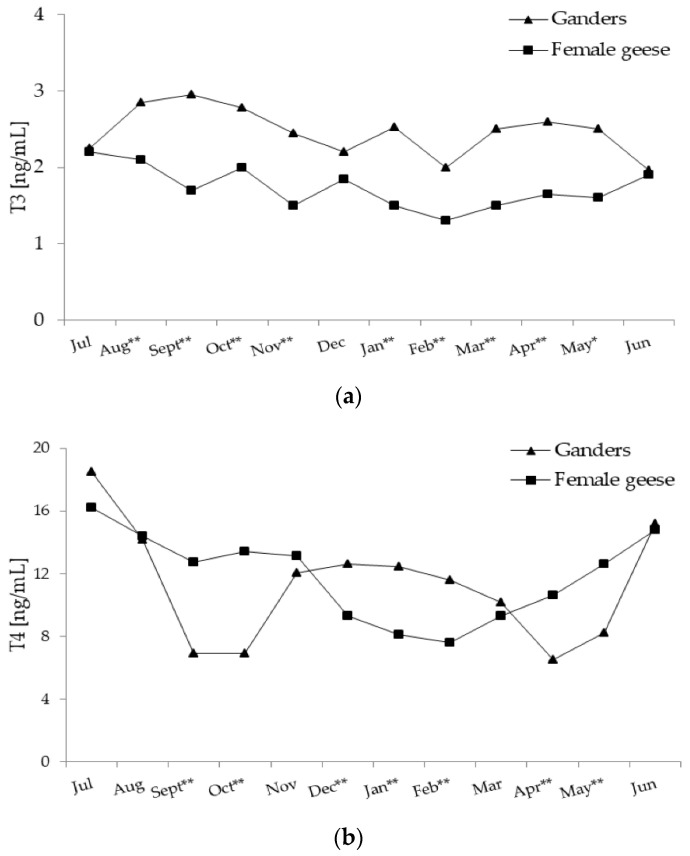
(**a**) Plasma concentration of T3 (tiiodothyronine) in 2-year-old Zatorska ganders (*n* = 10) and female geese (*n* = 19) during the annual period. Breeding flock (10 ganders and 40 female geese; natural mating). Reproduction period: January–June. Photoperiod during reproduction: short-day (SD) 10L:14D. Data are means presented in monthly intervals. Annual pattern of T3 in ganders fit a linear trend (*p* < 0.01) and in female geese random fluctuation (no trend) was noted. The months with different superscripts indicate significant differences between means for sex—ganders vs. female geese (* *p* < 0.05; ** *p* < 0.01). (**b**) Plasma concentration of T4 (thyroxine) in 2-year-old Zatorska ganders (*n* = 10) and female geese (*n* = 19) during the annual period. Annual pattern of T4 in ganders fit a quartic trend (*p* < 0.01) and in female geese fit a quadratic trend (*p* < 0.01). The months with different superscripts indicate significant differences between means for sex—ganders vs. female geese (* *p* < 0.05; ** *p* < 0.01).

In ganders, annual changes in the T3 concentration were characterised by a significant (*p* < 0.01) negative linear trend with a slow decrease from August until June (Figure 3a). In female geese, annual T3 concentrations randomly fluctuated without any significant trend. 

During most of the annual period, T3 concentration was higher (*p* < 0.01; in May *p* < 0.05) in ganders than in female geese. Only in July, December and June were concentrations of T3 similar in both sexes. A significant interaction (*p* < 0.01) between the sex and month factors during the annual period was demonstrated.

##### T4 Concentration

In ganders, annual T4 concentration pattern fit a significant (*p* < 0.01) quartic trend (Figure 3b), which was concave upward then downward and then again upward. In the period from July–September, T4 concentration decreased and remained low until October and then increased to a moderate level during the period from November–January. From February another decrease was observed to a low level in April which then increased again. In female geese, annual changes in the T4 concentration were characterised by a significant (*p* < 0.01) quadratic trend with a concave upward pattern. A gradual decrease in T4 during the period from July–February was evaluated and then from March a slow increase to a high level in June was observed.

During the periods from September–October and from April–May T4 concentrations were higher (*p* < 0.01) in female geese compared to ganders. On the contrary, during the period from December–February concentration of T4 was higher (*p* < 0.01) in ganders than female geese. A significant interaction (*p* < 0.01) between the sex and month factors during the annual period was demonstrated.

#### 3.2.3. Gonadal Steroid Hormones Concentration—T, E2, P4 

##### T Concentration 

**Figure 4:**
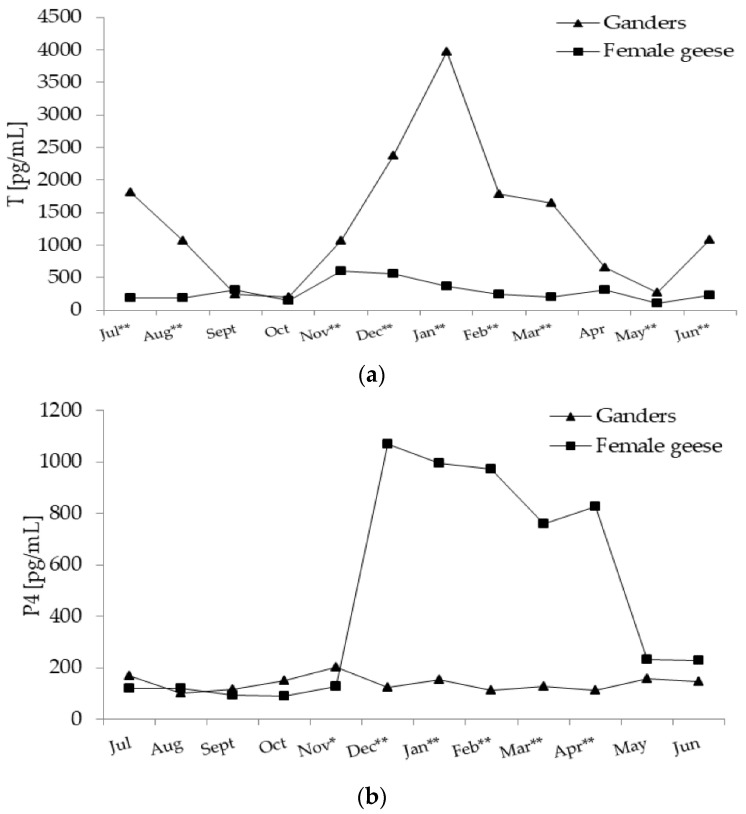

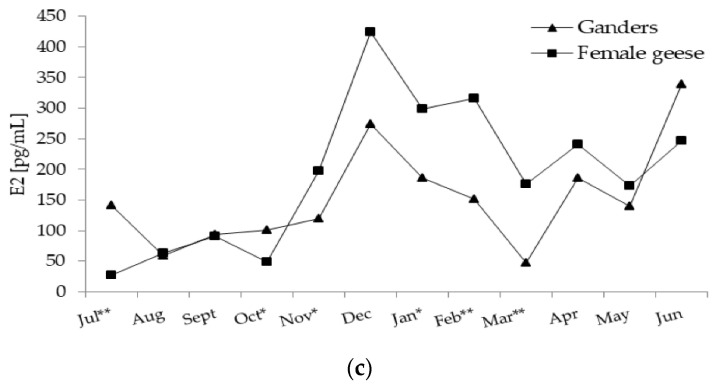
(**a**) Plasma concentration of T (testosterone) in 2-year-old Zatorska ganders (*n* = 10) and female geese (*n* = 19) during the annual period. Breeding flock (10 ganders and 40 female geese; natural mating). Reproduction period: January–June. Photoperiod during reproduction: short-day (SD) 10L:14D. Data are means presented in monthly intervals. Annual pattern of T in ganders fit a cubic trend (*p* < 0.01) and in female geese fit a quadratic trend (*p* < 0.01). The months with different superscripts indicate significant differences between means for sex—ganders vs. female geese (* *p* < 0.05; ** *p* < 0.01). (**b**) Plasma concentration of P4 (progesterone) in 2-year-old Zatorska ganders (*n* = 10) and female geese (*n* = 19) during the annual period. Annual pattern of P4 in ganders presented random fluctuation (no trend) and in female geese fit a quadratic trend (*p* < 0.01). The months with different superscripts indicate significant differences between means for sex—ganders vs. female geese (* *p* < 0.05; ** *p* < 0.01). (**c**) Plasma concentration of E2 (estradiol) in 2-year-old Zatorska ganders (*n* = 10) and female geese (*n* = 19) during the annual period. Annual pattern of E2 in ganders fit a cubic trend (*p* < 0.01) and in female geese fit a quadratic trend (*p* < 0.01). The months with different superscripts indicate significant differences between means for sex—ganders vs. female geese (* *p* < 0.05; ** *p* < 0.01).

In ganders, annual changes in T concentration were characterised by a significant (*p* < 0.01) cubic trend, which was concave upward followed by concave downward (Figure 4a). From July–October, T level decreased and then from November onward, increased to reach peak level in January and then decreased again to the lowest level in May. In female geese the annual T concentration pattern fit a significant (*p* < 0.01) quadratic trend, which was concave downward. In the period from July–November, T increased and then from December slowly decrease to a low value in April. 

During most of the annual period, T concentration was higher (*p* < 0.01) in ganders then in female geese. Only in September, October, and April was T concentration similar in both sexes. A significant interaction (*p* < 0.01) between the sex and month factors during the annual period was demonstrated.

##### P4 Concentration 

In ganders annual P4 concentration pattern randomly fluctuated without any significant trend. In female geese annual P4 concentration pattern fit a significant (*p* < 0.01) quadratic trend (Figure 4b), which was concave downward. A stable level was noted in the period from July–November which then sharply increased during December. The high level of P4 was evaluated until April and then in May it decreased sharply to a low level. 

During the November (*p* < 0.05)–April period P4 concentration was higher (*p* < 0.01) in female geese then ganders. A significant interaction (*p* < 0.01) between the sex and month factors during the annual period was demonstrated.

##### E2 Concentration 

In ganders, annual E2 concentration pattern fit a significant (*p* < 0.01) cubic trend (Figure 4c) which was concave downward followed by concave upward. In August, E2 level increased to a high level in December and then decreased to a low level in March and then re-increased to the highest level in June. In female geese, annual changes in the E2 concentration were characterised by a significant (*p* < 0.01) quadratic trend which was concave downward. In the period from July–October, E2 concentration was at a low level and then increased from November to a peak level in December and then to a stable level in the January–February period which then slowly decreased until June.

In July (*p* < 0.05) and October (*p* < 0.01) E2 concentration was higher in ganders compared to female geese. While in November (*p* <0.05), January (*p* <0.05) and the February–March (*p* < 0.01) period E2 level was higher in female geese than ganders. A significant interaction (*p* < 0.01) between the sex and month factors during the annual period was demonstrated.

### 3.3. Coefficients of Correlation between Hormone Concentrations 

The PRL concentration was negatively correlated to T3 and T in both sexes (Table 3). Moreover, negative correlations between PRL and T4 in ganders and P4 and E2 in female geese were noted. However, in female geese, positive coefficient of correlation values was noted between PRL and T4. As for THs hormones in ganders, T3 was negatively correlated to T4 and T4 was positively correlated with T and P4. In female geese, T4 correlated negatively with T and positively with P4. Moreover, for ganders, positive correlations between T and P4 and E2 were noted. In female geese, gonadal steroid hormones correlated positively with each other. Most of the listed correlations were low, but statistically significant.

## 4. Discussion

Domestic, long-day breeding Zatorska geese kept in a controlled environmental condition presented: (a) elevated plasma PRL levels post-breeding and during the second half of the breeding–laying period in both sexes, (b) relatively low sex-related differences in PRL but higher levels were noted in ganders by the end of the breeding–laying period and in female geese in the post-breeding period, (c) elevation in plasma PRL levels by the end of the breeding–laying period were detected earlier in ganders than in female geese, (d) a linear trend for the annual pattern of T3 in ganders and no trend for female geese, (e) higher magnitudes of variation in annual T4 levels in ganders than in female geese but in both sexes a higher level was noted by the end of the laying and post-breeding periods, (f) annual patterns of THs were partially in agreement with existing theories in free-living birds with a permissive but not causal role of THs in termination of breeding, (g) annual quadratic trends for T, P4, and E2 patterns in female geese associated with gonad redevelopment and regression, and (h) annual changes in T and E2 in ganders suggested that they terminate their breeding–laying period earlier than females, as indicated by previous fertility results.

Hormonal similarity between sexes is important in domestic geese, which are kept only in multi-male and multi-female social structures [30]. In the present study, the classic concept of hormonal co-variation between pair partners affecting the reproductive success in monogamous birds [22] was not analysed. In the conditions of poultry production, it is not possible for female geese to perform natural incubation and rearing chicks. Moreover, in a polygynous flock situation the mate may adapt its hormone levels to one exclusive partner or to multiple partners in response to social context [20].

The breeding–laying period of Zatorska geese occurs between January and June, and egg production was at the rate of 30%. These results are consistent with previous study for 2-year-old geese [11], suggesting suitability for studying the endocrine pattern during an annual period. 

The ELISA method applied in this study has been used for the determination of the levels of plasma gonadal steroid hormones and PRL for several years in the laboratory of the Department of Animal Science of The Hebrew University of Jerusalem in Israel. Until now, it has been used for the evaluation of gonadal steroids in plasma of domestic turkeys [31] and these hormones and PRL in laying [32] and broiler breeder chickens [33,34] and domestic geese [26,27].

We showed that in seasonal Zatorska geese, plasma PRL level was high during the post-breeding and the second half of the breeding–laying periods and low during pre-breeding and the onset of laying. Those results are partially in agreement with the previous study [13] reported on European geese kept in natural photoperiod environments. Similar to Péczely et al. [13], higher levels of PRL in female geese in the post-breeding period than in ganders was reported. This may be explained by the fact that by the end of the breeding–laying period some geese created natural nests. In chickens [32] and geese [35], incubation behaviour is related to plasma PRL elevation. Contrary to presented results, in long-day breeding geese Yang et al. [14] observed that PRL level was the highest during the peak of egg production. On the other hand, in short-day breeders [17,18,19] seasonal patterns of PRL levels were opposite to those of gonadal steroids with low values in the breeding season and high values during non-breeding. An elevated PRL level could play a role in linking the end of reproduction and the start of the moult [23,36]. Interestingly, in this study, increases in PRL levels by the end of the breeding–laying period were noted earlier in ganders then in female geese. It suggests that the breeding–laying period of ganders may be shorter than that of female geese. This view finds support from our previous observation that during the spring period (April), a reduction in fertility in a flock of Zatorska geese was connected with a decrease in the frequency of mating activity [11] and negative changes in semen parameters [26]. In free-living mute swans, Dawson et al. [37] found no difference in the timing or magnitude of changes in PRL level between the male and female of non-breeders but in pairs, changes in PRL and the onset of moult were delayed in males relative to females. In natural breeding conditions the presence of chicks appears to have stimulated PRL secretion, and presumably, this coincided with a change in behaviour of males from sexual/territorial to parental/defensive [37]. In commercial geese artificial incubation of breeding flocks is common so there is no such type of stimulation of male behaviour.

In this research, PRL levels gradually increased before the onset of sexual redevelopment in both sexes and was relatively high by the end of egg laying when eggs are still produced. Moreover, at onset of laying, all classes of follicles in ovary and the largest weight of testes compared to non-breeding stage were noted. It looks like PRL below a threshold concentration appears to be also pro-gonadal in domestic geese. This view finds support in the study by Ma et al. [38] in which the PRL plays a stimulatory role in the SWF to SYF transition in geese. It is also found that immunisation of aging roosters against VIP depressed reproductive activity and PRL administration restored it [39]. Thus, it cannot be ruled out that in domestic geese, PRL is a potential regulator in both the initiation and the termination of the breeding period.

There is also evidence that the post-breeding moult is governed by changes in THs [7] and so the circulating level may act with other hormones to modulate reproduction activity. In the present research, plasma T3 level was higher in ganders than in female geese during most of the annual period. This may suggest sex-related differences in basal metabolic rate. In ganders, a slow, linear decrease in T3 level with the progression of the reproduction season was observed but surprisingly in female geese random variation was shown. The level of T4 starts to increase in the breeding–laying period earlier in female geese, and a higher level at the second half of breeding than in ganders was noted. However, in both sexes, a higher level of T4 was noted by the end of breeding and in the post-breeding period suggesting onset of natural moult. In domestic chickens [40] a negative relationship between circulating THs and ovarian function during sexual maturity exists. Also, in free-living Humboldt penguins peak of T3 and T4 levels during the annual period overlapped with moulting when the gonadal steroids are low [41]. Present results partially correspond with obtained by Péczely et al. [7,13] in which THs were relatively low in the spring but increased to high levels in the summer. More recently Zhu et al. [16] reported that patterns of THs levels in long-day breeding Chinese ganders changed in a reciprocal fashion to that of T. On the other hand, in female geese [15] no relationship was found between T4 and reproductive activities, but T3 levels were high under initial reproduction and photorefractoriness. However, it must be emphasised that in research in short-day breeding Magang ganders [17], there was no marked seasonal differences in T3 level. THs are well-known as being hormones with multiple actions so their level can also change in annual period in respect to factors that are not linked to moult such as metabolic needs. Moreover, the effect of THs on reproduction activity my not only depend on circulating levels but also on the location and abundance of receptors. In domestic hens, THs can affect the function of the ovary through TRα and TRβ which are shown in all compartments of the ovary [42]. Therefore, it is not very surprising that the results of presented research are somewhat inconsistent. These data are partially in agreement with existing theories in free-living birds on the specific role of THs in moult and also on termination of breeding, which is rather permissive than causal [6].

Annual pattern variations in plasma gonadal steroids in Zatorska geese are associated with the reproductive status of birds and with the redevelopment and regression of gonads. Morphometric analyses revealed that testes and ovary undergo marked changes between peak-breeding activity and non-breeding phases. These findings correspond with previous histology of testes in Zatorska ganders [27] and study of ovary in Magang geese [18]. 

Testosterone in ganders was at a medium level in the post-breeding period and then increased during the transition from the pre-breeding to the onset of laying period and decreased with the decline in the egg-laying rate. These findings correspond with studies by Hirschenhauser et al. [12] and Péczely et al. [7,13] in which high levels of T were evaluated in spring and autumn periods but were low in summer. The seasonal changes in T levels are also typical for long-day breeding Yangzhou [16] and short-day Magang ganders [17]. During most of the annual period, T concentration was about five-fold higher in ganders than in female geese. Also, in female geese increases in T level in the pre-breeding period were noted but decreases were observed before onset of the breeding–laying period. In free-living female birds of seasonal reproduction T levels fluctuate during the annual period [43] with the peak during periods of territorial establishment or mate acquisition and decline as parental demands increase. In domestic chicken females, T levels change during the period before sexual maturation implying a role for T in the maturation of the reproductive system [44]. Furthermore, changes in the level of T in the ovulation cycle are observed. Both prehierarchical and hierarchical follicles in the ovary express an androgen receptor, and androgens regulate steroidogenesis via a paracrine and/or autocrine action [45]. Testosterone directly stimulates granulosa cell P4 production as well as LH receptor mRNA expression of preovulatory, hierarchical follicles [46]. It is noteworthy that, in the present study, the same low level of T in both sexes was evaluated in the second half of the breeding period (spring). Additionally, in ganders at this time, sharp increases in PRL levels were noted.

In ganders, annual P4 concentration pattern fluctuated randomly while in female geese was at a high level in the breeding–laying period and decreased by the end of egg production. Also, E2 level in female geese was at a high or medium level until the end of laying. In this study at the onset– and breeding–laying periods all groups of ovarian follicles were observed. In domestic hens the stroma, the white non-hierarchical and the smaller yellow hierarchical follicles (F6–F3) are a source of E2, while the largest yellow preovulatory follicles are a major source of P4 [47]. On the other hand, in ganders E2 level surprisingly decreased sharply in peak egg production and then increased. During pre-breeding and the first half of the breeding–laying period E2 level was higher in female geese than ganders but at the second half a similar level in both sexes was noted. Estrogens are involved in regulation of ganders’ reproduction and seasonal fluctuations in the expression of estrogen receptors (ERs) in the testes were noted [48]. Interestingly, Péczely et al. [7] reported changes in E2/T ratio in ganders with its increase connected with progress of moulting. Moreover, Péczely et al. [7] stated that the initiator effect of gonadal steroids on reproduction of geese was connected with essential drastic decreases at the start of moult. In this context it is important to note that PRL was correlated negatively with T in both sexes and with P4 and E2 in female geese. Everything considered it may be suggesting that the end of the breeding period of Zatorska goose seems to be about one month earlier in ganders.

In flocks of domestic geese production of hatching eggs with optimal fertility during breeding–laying period determines profitability. Previous studies indicate that modulation of PRL with dopamine agonist [49] and blockage of serotonin synthesis [32] may enhance the reproductive efficiency of domestic hens. Also, using different photoperiod stimulation in the breeding–laying period like in the study by Zhu et al. [15] may be considered in future work on Zatorska geese. 

## 5. Conclusions

The present study extended previous reports in Zatorska ganders concerning the endocrinology of reproduction and provides a complete data set of the changes in PRL, THs, and gonadal steroids during the annual cycle in control environmental conditions. We found marked variations and sex-related differences in the levels of PRL, THs, and gonadal steroids. More importantly, circulating PRL level, which is considered the most important in regulating seasonal changes in birds, increased earlier in domestic ganders then in female geese. Also, T level in ganders decreased and E2 increased in the second half of the breeding–laying period. Thus, it seems probable that the breeding period characterised by major endocrine axes terminated earlier in ganders. This points to the importance of searching for methods of extending the breeding through endocrine axis or photoperiod manipulations to improve synchrony of both sexes. Improving reproduction results is a priority in the current goose breeding industry. 

## Figures and Tables

**Table 1:** Body weight and testes characteristics (median, interquartile range) in 2-year-old Zatorska ganders (*n* = 5/stage) during the different stages of the annual period.

Stage	Body Weight (g)		Testes											
			Right		Left		Right		Left		Right		Left	
			Weight (g)				Major Axis (mm)				Minor Axis (mm)			
Post-breeding (July) *	6231 ^ab^	380	4.0 ^ab^	2.2	4.9 ^ab^	1.2	24.9 ^ab^	2.1	28.7 ^ab^	3.3	19.0 ^b^	3.8	19.7 ^ab^	2.2
Non-breeding (September)	4970 ^b^	302	1.7 ^a^	0.7	1.7 ^a^	1.3	16.8 ^a^	1.1	18.6 ^a^	1.6	8.1 ^a^	1.6	9.1 ^a^	3.6
Pre-breeding (November)	4891 ^b^	955	3.8 ^ab^	1.8	7.2 ^ab^	3.7	22.8 ^ab^	1.1	29.8 ^ab^	2.1	14.5 ^ab^	0.6	19.5 ^ab^	1.9
Onset of laying (January)	6597 ^a^	174	6.8 ^b^	2.0	9.8 ^b^	2.7	27.0 ^b^	5.5	28.7 ^b^	3.5	18.9 ^b^	1.7	24.0 ^b^	1.1
Breeding–laying (April)	6235 ^ab^	734	4.3 ^ab^	0.4	8.2 ^b^	2.7	28.2 ^b^	0.8	33.2 ^b^	2.0	17.0 ^ab^	2.6	24.5 ^b^	2.0

* 50°03′ N/19°57′ E; Reproduction period: January–June. Within columns, medians denoted by the different letter superscripts differ significantly ^a,b^ *p* < 0.05.

**Table 2:** Body weight, ovary and oviduct characteristics (median, interquartile range) in 2-year-old Zatorska female geese (*n* = 5/stage) during the different stages of the annual period.

Stage	Body Weight (g)		Ovary								Oviduct			
			Weight (g)		Follicles						Weight (g)		Lenght (cm)	
					LWF: 3–8 mm (*n*)		SYF: 9–18 mm (*n*)		LYF: 19–64 mm (*n*)					
Post-breeding (July) *	5405 ^ab^	398	7.5 ^ac^	1.3	7 ^a^	7	-	-	-	-	16.6 ^ab^	2.1	50.0 ^abc^	9.0
Non-breeding (September)	4870 ^b^	300	1.1 ^a^	0.4	25 ^ab^	8	-	-	-	-	9.3 ^a^	3.7	29.0 ^a^	6.5
Pre-breeding (November)	4770 ^b^	700	1.5 ^a^	0.4	31 ^ab^	4	-	-	-	-	9.3 ^a^	2.4	33.0 ^ab^	6.0
Onset of laying (January)	5698 ^a^	375	239.5 ^b^	32.0	39 ^b^	8	8 ^a^	1	8 ^a^	1	78.9 ^b^	14.4	114.5 ^c^	4.0
Breeding–laying (April)	5709 ^a^	662	127.8 ^bc^	5.6	33 ^b^	8	6 ^a^	5	5 ^b^	1	93.5 ^b^	5.1	111.5 ^bc^	1.5

* 50°03′ N/19°57′ E; Reproduction period: January–June. *n*–number of follicles; LWF–large white follicle; SYF–small yellowish follicle; LYF–large yellow follicle. Within columns, medians denoted by the different letter superscripts differ significantly ^a,b,c^ *p* < 0.05.

**Table 3:** Pearson (r_p_) phenotypic correlation coefficient for plasma concentration of PRL (prolactin), thyroid hormones (T3–triiodothyronine, T4–thyroxine) and gonadal steroid hormones: T (testosterone), P4 (progesterone), E2 (estradiol) in 2-year-old Zatorska ganders (*n* = 10) and female geese (*n* = 19) during the annual period.

		Ganders					
		PRL [ng/mL]	T3 [ng/mL]	T4 [ng/mL]	T [pg/mL]	P4 [pg/mL]	E2 [pg/mL]
**Female geese**	PRL [ng/mL]	-	r_p_ = −0.133 *	r_p_ = −0.129 *	r_p_ = −0.382 ***	r_p_ = −0.116	r_p_ = 0.020
	T3 [ng/mL]	r_p_ = −0.117 *	-	r_p_ = −0.228 ***	r_p_ = −0.032	r_p_ = −0.122	r_p_ = −0.039
	T4 [ng/mL]	r_p_ = 0.244 ***	r_p_ = 0.013	-	r_p_ = 0.436 ***	r_p_ = 0.230 ***	r_p_ = 0.105
	T [pg/mL]	r_p_ = −0.334 ***	r_p_ = 0.107	r_p_ = −0.213 ***	-	r_p_ = 0.164 **	r_p_ = 0.141 *
	P4 [pg/mL]	r_p_ = −0.143 **	r_p_ = 0.073	r_p_ = 0.129 *	r_p_ = 0.233 ***	-	r_p_ = 0.048
	E2 [pg/mL]	r_p_ = −0.409 ***	r_p_ = 0.108	r_p_ = −0.118 *	r_p_ = 0.284 ***	r_p_ = 0.445 ***	-

Correlation is significant at the * *p* < 0.05, ** *p* < 0.01, *** *p* < 0.001 level.

## Data Availability

The data presented in this study are available on request from the corresponding author.
